# An Atypical Adult Presentation of Avoidant/Restrictive Food Intake Disorder

**DOI:** 10.7759/cureus.94475

**Published:** 2025-10-13

**Authors:** Alahmed Salah Saleh Ahmed, Rabindara Katwal

**Affiliations:** 1 Internal Medicine, Portsmouth Hospital University NHS Trust, Portsmouth, GBR; 2 Acute Medicine, Portsmouth Hospital University NHS Trust, Portsmouth, GBR

**Keywords:** arfid, avoidant/restrictive food intake disorder, eating behaviors, eating habits, gelatinous bone marrow transformation, gelatinous transformation of bone marrow, malnutrition, picky eating, serous marrow atrophy, zinc deficiency

## Abstract

Avoidant/restrictive food intake disorder (ARFID) is a rare eating disorder usually seen in children and adolescents; adult presentations are uncommon.

We report a 46-year-old male patient with generalized rash, weight loss, and weakness. He underwent extensive investigations, including MRI, CSF, CT, PET-CT, bone marrow biopsy, and autoimmune/paraneoplastic screening, due to diagnostic uncertainty and the non-specific presentation.

A multidisciplinary team identified ARFID related to disordered eating behaviors (rushed eating, fear of fullness). The diagnosis was based on the history taken from the patient about his eating habits and the absence of any other conditions that could explain the symptoms. He was managed with nasogastric feeding, nutritional supplementation, dietetic input, and psychiatric support, which led to symptom improvement and steady weight gain.

This case illustrates an atypical adult presentation of ARFID with systemic manifestations, emphasizing the need for multidisciplinary recognition and treatment.

## Introduction

Avoidant/restrictive food intake disorder (ARFID) is a relatively recently identified eating disorder that was included in the Diagnostic and Statistical Manual of Mental Disorders, Fifth Edition (DSM-5) in 2013. It is characterized by restrictive eating patterns that can lead to weight loss, nutritional deficiencies, and significant psychological and physical impairment, often requiring reliance on supplements or enteral feeding. Importantly, these features occur in the absence of body image disturbance, which distinguishes ARFID from eating disorders such as anorexia nervosa, where there is a fear of becoming fat, or bulimia nervosa, where there are episodes of recurrent binge eating and compensatory mechanisms such as induced vomiting and the use of laxatives [1].

ARFID is most commonly diagnosed in children and adolescents; however, it can present in adults and lead to significant clinical complications. There are no clear or accurate data outlining the prevalence of the condition in adults, mainly due to the lack of large-scale studies with validated measures [2]. ARFID prevalence is similar in both sexes, unlike other eating disorders, which are more prevalent in the female population [2]. Subtypes of ARFID include avoidance due to sensory sensitivities, fear of adverse consequences (such as choking), and lack of interest in food [3]. The disorder is frequently associated with other conditions such as autism spectrum disorder and attention-deficit/hyperactivity disorder (ADHD) [4].

Early recognition of ARFID is critical to prevent complications such as severe malnutrition, impaired growth, and psychosocial dysfunction. Nonetheless, diagnosis can be challenging due to symptom overlap with other disorders, subtle clinical presentation, and limited clinician awareness [5]. Literature on ARFID, particularly in adults, remains scarce as the disorder has only recently been formally recognised.

This report describes a case of ARFID diagnosed for the first time in adulthood, highlighting its diagnostic challenges, clinical features, and treatment.

## Case presentation

A 46-year-old man presented to the emergency department with one day of knee pain. He had no features suggestive of septic arthritis. Blood investigations were unremarkable for infection but revealed normocytic anaemia and mild hyponatraemia. Vital signs were within normal limits. He was referred to the Same Day Emergency Care (SDEC) unit, where further history revealed unintentional weight loss, muscle wasting, and slurred speech progressing over the past year.

His past medical history was unremarkable apart from shingles three weeks prior. He was only taking folic acid supplementation. There was no significant family history, and he followed a non-restrictive diet (not vegan or vegetarian).

On examination, the patient appeared cachectic with marked muscle wasting, hypophonia, and dysarthria. A generalized rash was present on all limbs, with peripheral oedema. Neurological examination showed mild weakness of the hands, fatigability of the ocular muscles, and proximal weakness in the shoulders. Given the constellation of findings, paraneoplastic syndrome and neuromuscular disease were considered. He was therefore investigated extensively with imaging, autoantibody panels, and bone marrow biopsy.

**Figure 1:**
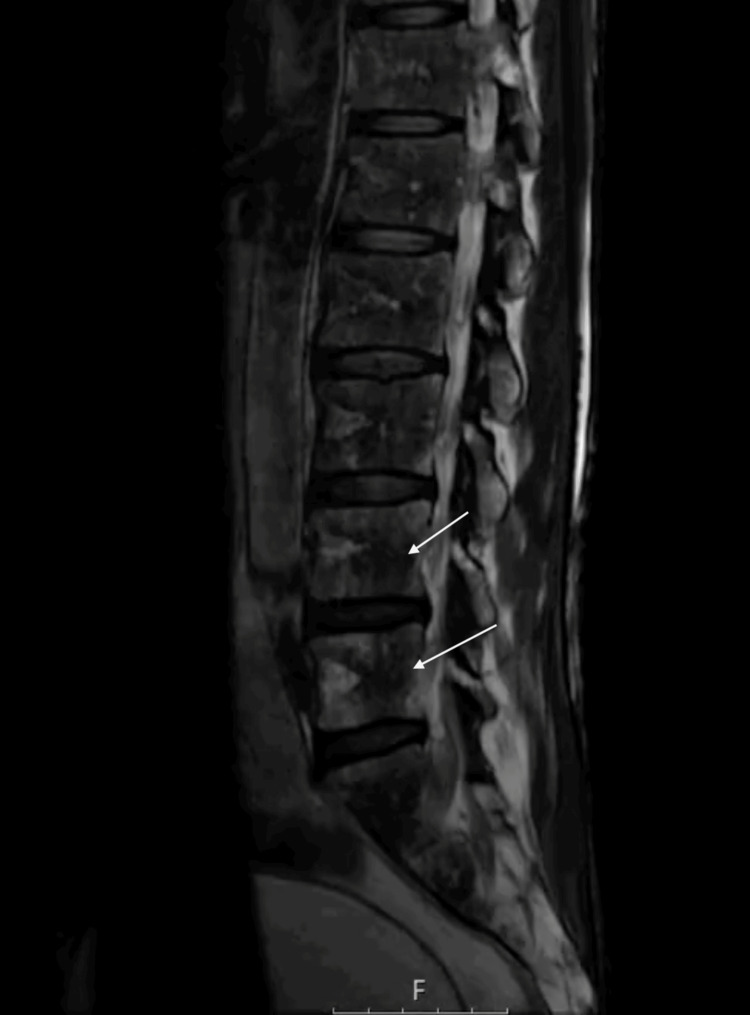
Sagittal T1-Weighted MRI of the Lumbar Spine Sagittal T1-weighted MRI of the lumbar spine demonstrating diffuse low marrow signal intensity across multiple vertebral bodies, reflecting serous (gelatinous) marrow atrophy.

**Figure 2:**
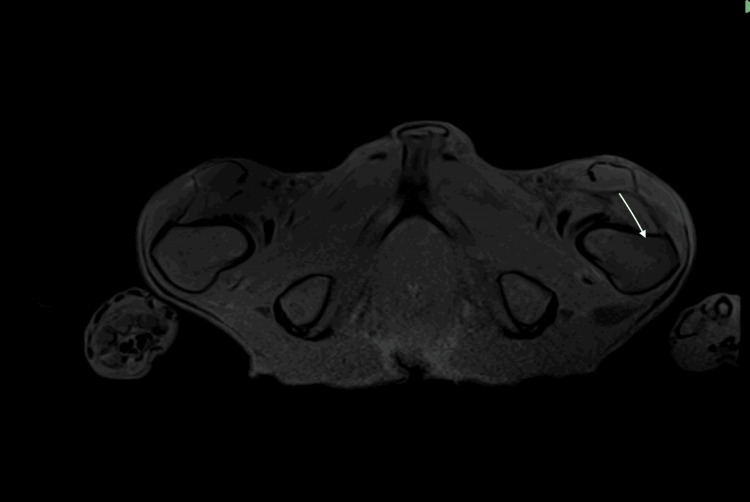
MRI of the Pelvis MRI of the pelvis demonstrating serous marrow atrophy, with diffuse loss of normal fatty marrow signal in the pelvic bones and proximal femora. The surrounding musculature appears preserved, with no fatty infiltration or inflammatory change.

**Figure 3:**
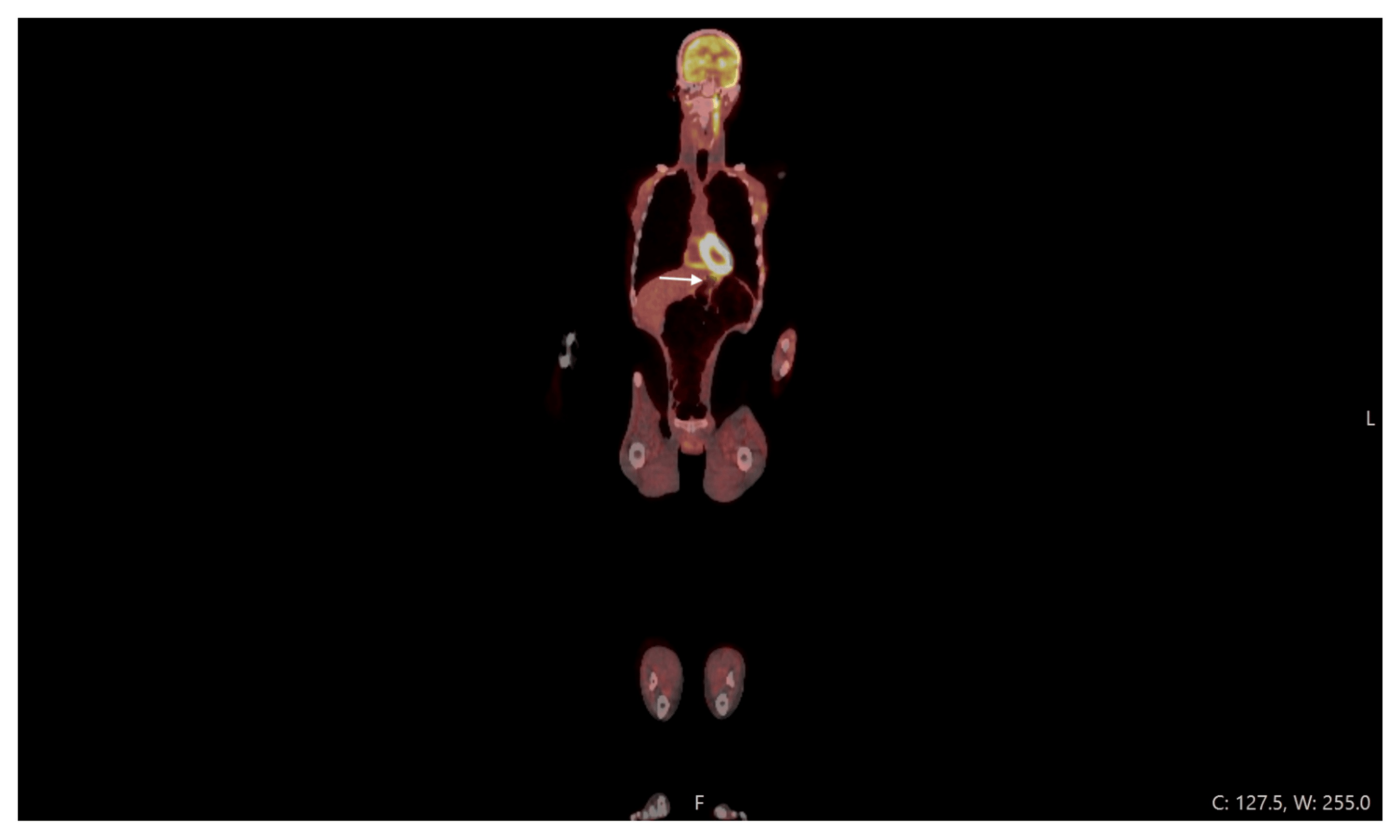
PET CT scan Diffuse gastric uptake (likely incidental finding).

Investigations demonstrated bony lesions consistent with insufficiency fractures and markedly hypocellular bone marrow. MRI of the brain showed diffuse marrow signal abnormalities with dural enhancement, raising the possibility of hematologic or metastatic disease. Additionally, an MRI of the lumbar spine showed low marrow signal in multiple vertebrae (Figure 1), and an MRI of the pelvis showed serous marrow atrophy in the pelvic bones and proximal femora, with normal surrounding muscles preserved (Figure 2). PET-CT confirmed multiple fractures but no malignancy, and also demonstrated diffuse gastric uptake consistent with inflammatory changes, likely an incidental finding (Figure 3). MRI of the lower limbs revealed serous bone marrow atrophy, a non-neoplastic transformation associated with chronic illness and poor nutritional status. Bone marrow biopsy confirmed gelatinous transformation. A summary of key investigations is provided in Table 1.

Extended autoimmune, paraneoplastic, and infectious screens were negative, including Lyme and syphilis serologies. Although the anti-Jo1 antibody was positive and creatine kinase (CK) was initially elevated at 1300 µmol/L, there were no clinical features of myositis, and CK normalised spontaneously without treatment. Zinc levels were significantly reduced. Nerve conduction studies and CSF analysis were normal (Table 2).

**Table 1:** Key investigations

Investigation	Findings
MRI brain & spine	Diffuse bone marrow changes with mild dural enhancement; no intracranial or cord pathology
CT chest/abdomen/pelvis	Marked cachexia; no measurable malignancy
PET-CT	Multiple insufficiency fractures; diffuse gastric uptake, likely inflammatory gastritis
MRI lower limbs	Serous atrophy of bone marrow; no muscle fatty, fibrous, or inflammatory change
Nerve conduction studies	Normal
Bone marrow biopsy	Hypocellular marrow with gelatinous transformation, consistent with severe malnutrition

**Table 2:** Key Lab Results GAD: glutamic acid decarboxylase; CSF: cerebrospinal fluid

Test	Value	Reference Range
Hb	107 g/L	130–180 g/L
Zinc	5.8 Umol/L	11-24 Umol/L
GAD autoantibodies	<0.5 u/ml	0–5 u/ml
Acetylcholine receptor antibody	0.30 nmol/L	0–0.89 nmol/L
Anti-skeletal muscle	Negative	Negative
Oligocolonal banding CSF	Negative	Negative
Syphilis antibody test	Negative	Negative

Imaging and investigations primarily revealed severe bone and marrow changes, which affected the patient’s ability to mobilise normally and safely. He experienced multiple inpatient falls during his admission and required a Zimmer frame for mobility.

Given severe malnutrition, a nasogastric tube was inserted, and nutritional blood work confirmed multiple micronutrient deficiencies. Enteral feeding was introduced cautiously to prevent refeeding syndrome, alongside oral nutritional supplements and vitamin D replacement. Weight monitoring was initiated, and the mental health team reviewed the patient due to suspicion of an underlying psychological disorder contributing to dietary restriction. Further history and collateral information revealed longstanding eating behaviours consistent with ARFID, including discomfort with postprandial fullness and rushing through meals.

The diagnosis was established due to the absence of any other medical or psychological condition that could account for the patient’s presentation. Furthermore, the clinical features were consistent with the DSM-5 criteria for avoidant/restrictive food intake disorder, which describe an eating disturbance such as limited interest in food, avoidance based on sensory characteristics, or concern about aversive consequences of eating. This disturbance results in a persistent failure to meet nutritional and energy requirements, leading to significant weight loss, nutritional deficiencies, dependence on enteral feeding or supplements, or marked impairment in psychosocial functioning [1].

**Figure 4:**
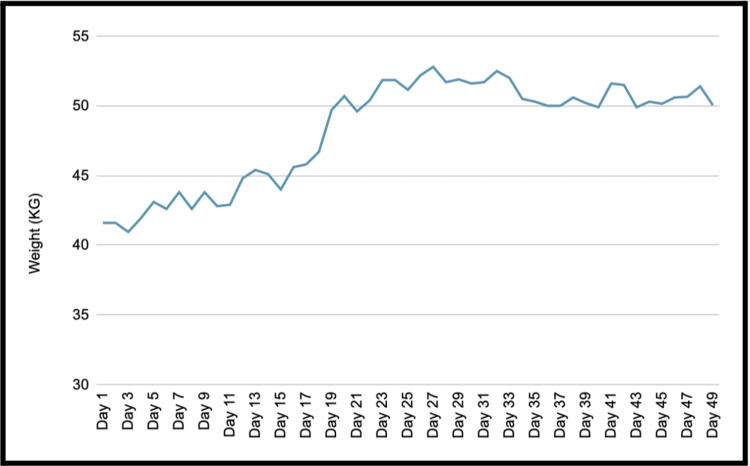
Weight measurements showing steady weight gain following enteral

The patient demonstrated a steady improvement in weight and functional status. After an initial rise, his weight plateaued at around 50-52 kg, where it remained stable (Figure 4). His creatine kinase normalised, and he regained muscle strength and energy (Table 3). Following a multidisciplinary review, he was discharged with community dietetic and psychiatric follow-up.

**Table 3:** Haemoglobin and Vit D three month after treatment.

Test	Value	Reference Range
Hb	135 g/L	130- 170 g/L
Vitamin D	92 mmol/L	50-300 mmol/L

## Discussion

ARFID is a relatively new diagnostic entity, first introduced in the fifth edition of the DSM-5 in 2013. It is defined by restrictive eating behaviours that lead to significant weight loss, nutritional deficiency, or psychosocial impairment, in the absence of body image disturbance that characterises other eating disorders, such as anorexia nervosa or bulimia nervosa. ARFID is most frequently described in children and adolescents, while adult presentations are rare and often under-recognised [1]. This is due to the lack of large-scale prevalence studies in the adult population [2].

This case illustrates an atypical adult presentation of ARFID with profound multisystem complications. The patient presented with severe malnutrition, unintentional weight loss, and functional decline. The initial clinical picture was confounded by additional findings of dysarthria, hypophonia, rash, and muscle weakness, raising concerns for neuromuscular disease, paraneoplastic syndrome, or autoimmune pathology. Differential diagnoses considered included motor neuron disease, inflammatory myopathies, and Whipple’s disease. The presence of a positive anti-Jo1 antibody and transient CK elevation further complicated the clinical picture, although the absence of muscle inflammation, rapid spontaneous CK normalisation, and lack of other systemic features argued against myositis.

The patient’s generalised rash was later attributed to zinc deficiency, a recognised cutaneous manifestation of micronutrient deficiency in malnutrition. Zinc deficiency can cause dermatitis, often acral and periorificial, but may also present with widespread involvement, as in this case [3]. The recognition of this cutaneous finding was an important clinical clue supporting a nutritional aetiology rather than a primary dermatological or autoimmune disorder.

Extensive investigations excluded autoimmune, infectious, and malignant conditions. Imaging demonstrated multiple insufficiency fractures and marrow signal abnormalities. MRI of the lower limbs revealed serous atrophy of bone marrow, a rare but characteristic finding of chronic illness and severe malnutrition. Bone marrow biopsy confirmed gelatinous transformation, also referred to as “starvation marrow,” which has been described in association with anorexia nervosa and other wasting conditions [4]. These changes represent a severe depletion of fat and haematopoietic tissue, replaced by a gelatinous substance rich in hyaluronic acid. The finding in this case underscores the severity and chronicity of the patient’s nutritional deficiency.

Bone health complications, including insufficiency fractures, are recognised consequences of malnutrition and low body mass index. Prolonged nutrient deficiency leads to sarcopenia, osteopenia, and ultimately bone fragility [5]. In this case, multiple fractures were identified incidentally on imaging, despite the absence of acute trauma. The constellation of fractures, marrow changes, and micronutrient deficiencies highlighted the systemic impact of prolonged undernutrition.

Psychiatric evaluation revealed longstanding restrictive eating behaviours consistent with ARFID, including discomfort with postprandial fullness and rapid, rushed eating habits that limited nutritional intake. These maladaptive behaviours, rather than body image disturbance, were the key drivers of the patient’s malnutrition, aligning with DSM-5 diagnostic criteria [1]. The patient had no known neurodevelopmental disorder, but an outpatient assessment was arranged following discharge, as such evaluations can be unreliable during acute malnutrition.

The patient required a prolonged two-month hospital admission for nutritional stabilisation and recovery. This prolonged stay reflects both the severity of his presentation and the diagnostic challenges encountered, as ARFID is not well recognised in adult populations. Increased awareness of ARFID among clinicians may help reduce diagnostic delays and hospitalisation times in future cases.

The diagnostic process in this case was challenging, in part because ARFID is often overlooked outside of child and adolescent psychiatry. Furthermore, the broad systemic manifestations of malnutrition can mimic autoimmune, infectious, or malignant disease. Positive serological findings, such as anti-Jo1 antibody, may represent incidental findings that complicate the clinical picture. This emphasises the importance of careful clinicopathological correlation and avoidance of over-interpretation of isolated antibody results.

Management of ARFID requires a multidisciplinary approach. In this case, the patient’s care involved close collaboration between internal medicine, psychiatry, dietetics, and allied health professionals. Nutritional rehabilitation was central, with cautious introduction of nasogastric feeding to avoid refeeding syndrome, correction of micronutrient deficiencies, and ongoing oral supplementation. Psychiatric input was essential in addressing the behavioural drivers of restricted intake, while dietitians guided meal planning and weight restoration. The patient demonstrated steady weight gain and improved functional status, reflecting the effectiveness of integrated care.

Although nutritional and psychiatric follow-up was successful in improving symptoms in this case, a clear management approach for ARFID has not yet been established due to the novelty of the condition and the limited literature available. Some studies suggest that cognitive behavioural therapy (CBT) and family-based therapy (FBT) may be effective; however, these strategies have primarily been tested in children, and their effectiveness in adolescents and adults remains unclear [6].

Few adult case reports of ARFID describe such severe systemic manifestations, particularly serous marrow atrophy and insufficiency fractures. This case, therefore, adds to the growing recognition that ARFID can present in adulthood with significant medical sequelae. Clinicians should maintain a high index of suspicion for ARFID in adults presenting with unexplained weight loss and malnutrition, particularly when standard investigations fail to identify an organic cause. The use of screening tools such as the Nine Item ARFID Screen (NIAS) can be useful in the early recognition of the condition.

## Conclusions

This case describes an unusual adult presentation of ARFID with profound systemic consequences. It underscores the diagnostic complexity of ARFID in adulthood, highlights the risks of delayed recognition and prolonged hospitalisation, and reinforces the importance of multidisciplinary management for recovery. The use of screening tools such as the NIAS in primary care could support early diagnosis and help prevent severe complications. Awareness of the condition is also crucial, particularly as it is a newly identified disorder. Further large-scale studies are needed to determine the prevalence of ARFID, especially in adults, and to establish effective management strategies.
